# Pathogens with potential impact on reproduction in captive and free-ranging European bison (*Bison bonasus*) in Poland - a serological survey

**DOI:** 10.1186/s12917-021-03057-8

**Published:** 2021-11-04

**Authors:** Anna Didkowska, Daniel Klich, Anna Hapanowicz, Blanka Orłowska, Marta Gałązka, Magdalena Rzewuska, Wanda Olech, Krzysztof Anusz

**Affiliations:** 1 Department of Food Hygiene and Public Health Protection, Institute of Veterinary Medicine, Warsaw University of Life Sciences (SGGW), Nowoursynowska 166, 02-787 Warsaw, Poland; 2 Department of Animal Genetics and Conservation, Institute of Animal Sciences, University of Life Sciences (SGGW), Ciszewskiego 8, 02-786 Warsaw, Poland; 3 Department of Preclinical Sciences, Institute of Veterinary Medicine, Warsaw University of Life Sciences (SGGW), Ciszewskiego 8, 02-786 Warsaw, Poland

**Keywords:** *Chlamydia* spp., *Coxiella burnetii*, ELISA, European bison, *Leptospira interrogans*, *Neospora caninum*, reproductive system, serology, *Toxoplasma gondii*, zoonosis

## Abstract

**Background:**

The European bison is an endangered species, and as such it is extremely important to monitor herds for pathogens which can lead to reproductive failure. The aim of the present study was to determine the current prevalence of antibodies to pathogens known to potentially influence reproduction in European bison. Serum samples from 183 bison, originating from different parts of Poland, were tested using commercial ELISA tests for antibodies to *Chlamydia* spp., *Coxiella burnetti, Leptospira interrogans, Neospora caninum* and *Toxoplasma gondii*; the findings were compared between captive and main free-ranging herds, and with regard to the influence of demographic factors such as age and sex. The prevalence of seropositivity was also checked with regard to location and the animal species sharing it.

**Results:**

*Chlamydia* spp. antibodies were present in 48 out of 130 (36.9%) tested samples. *Coxiella burnetii* was found in one sample out of 178 (0.58%). *N. caninum* in 36 out of 172 (20.9%) and *T. gondii* in 23 out of 172 (13.4%). No sample was positive for leptospirosis. Neither sex nor age appeared to have a significant effect on the occurrence of antibodies to the identified species. The prevalence of *Chlamydia* spp. in the samples varied significantly according to location; however, similar frequency ranges were observed between free ranging and captive herds. In contrast, antibodies to *N. caninum* were more common in free-ranging herds than captive herds, with the highest frequency observed in the Bieszczady Mountains.

**Conclusions:**

*Chlamydia* spp., *N. caninum* and *T. gondii* might have a similar impact on the reproductive potential of European bison as they have on cattle. The high occurrence of antibodies to *N. caninum* in bison from the Bieszczady Mountains may be associated with the relatively high density of the wolf population in the area.

**Supplementary Information:**

The online version contains supplementary material available at 10.1186/s12917-021-03057-8.

## Background

The European bison (*Bison bonasus*) is a strictly-protected species which requires active conservation measures [1]. Following many years of hunting, leading nearly to its extinction, its population in Poland has been rebuilt since 1919 [2], and the country is currently home to 2269 individuals: 2048 in free-ranging herds and 221 in captive breeding [3]. However, its recovery continues [4, 5], and there is a growing need for effective health monitoring [1]. This is extremely important in European bison due to the relatively limited gene pool [6] and high susceptibility to infectious diseases [7], particularly considering the environmental threats it faces (see: [8, 9]).

Bovine tuberculosis (BTB) has had a considerable impact on the Polish bison population in recent years [10], and as such, the population is regularly monitored for BTB [11]. However, a number of other diseases also have zoonotic potential and should also be monitored. For example, chlamydiosis (chlamydophilosis), leptospirosis, Q fever and toxoplasmosis, all with zoonotic potential, have been identified in bison worldwide, and may pose a similar risk for reproduction as they do for cattle [12–14]. In addition, there is a need to monitor a number of other diseases that can have an adverse effect on the population size, such as brucellosis, by increasing the risk of abortions, stillbirths or weak calves [15], as well as those also that can impair male fertility, such as multi-etiological balanoposthitis, caused by *Corynebacterium* spp., *Pseudomonas aeruginosa, Escherichia coli, Fusobacterium necrophorum, Arcanobacterium pyogenes, Staphylococcus* spp. (coagulase negative), *Streptococcus* spp., *Onchocerca* spp., and *Trueperella pyogenes* [16, 17].

Chlamydial infections have been detected in over 450 species, including birds, mammals and humans [18]. It has recently been recommended that, due to high similarity of their genomes, the genera *Chlamydia* and *Chlamydophila*, within the family *Chlamydiaceae*, should be merged into a single genus *Chlamydia* [19]. In cattle, although chlamydial infection results in a variety of systemic symptoms, most concern the reproductive system [20]: chlamydiosis may lead to late abortion, as well as endometritis, vaginitis, seminal vesiculitis and weak calf births [21]. Infection by *Chlamydia abortus, Chlamydia suis* and *Chlamydia psittaci*, can lead to reproductive disorders, and all have been detected in Polish cattle herds [22].

Another disease which can lead to problems with reproduction is leptospirosis, caused by the bacterium *Leptospira*. The bacterium survives best in warm and wet conditions, and recent years have seen the disease extend its range, probably as a result of climate change and increasing globalization [23, 24]. Antibodies to *Leptospira* Bataviae, Bratislava, Canicola, Hardjo, Hebdomadis, Pomona, Cynopteri and Grippotyphosa have been detected in cattle in Poland [25]. Like *Chlamydia*, *Leptospira* can also have a negative impact on reproduction in cattle, causing abortions, embryonic deaths or infertility [26].

The most common symptoms of *Coxiella burnetii* infection in ruminants are abortion and stillbirth [27], and its main reservoirs are domestic ruminants, which spread the bacteria through urine, feces, milk, aminiotic fluid and placental tissue [28]. Ticks also play an important role in infection: twelve species are known to act as *C. burnetii* vectors [29], all of which have been found in Poland [30]. Three of them, *Ixodes persulcatus, Ixodes ricinus* and *Dermacentor reticulatus,* have been confirmed as ectoparasites in European bison [31]. Antibodies to *C. burnetti* have been found in 109 of 167 tested free-living species [32], including bison [16].

Bison reproduction can also be impaired by two parasitic diseases: neosporosis and toxoplasmosis. Regarding the former, *Neospora caninum* is recognized as an important agent causing abortion in cattle [13]; in addition, if the calf is born alive, there is a high probability of pelvic hyperextension and hydrocephalus, as well as neurological symptoms such as widespread ataxia [33]. The definitive hosts of *N. caninum* are dogs and some wild canids, which shed oocysts after eating the tissues of infected animals [34]. Toxoplasmosis, caused by *Toxoplasma gondii*, also has a potentially harmful effect on European bison reproduction*. T. gondii* oocysts are shed in the environment by the felid definitive hosts. Infected intermediate hosts may show neurological symptoms (canidae, pigs, poultry) or problems with the reproductive system (ruminants, pigs) [35].

Due to their close genetic relationship with cattle and the fact that they often share pastures, bison require monitoring for diseases that can be transmitted between the two species. Importantly, some of those etiological agents have zoonotic potential, and hence can represent public health threats through contact with infected animals or the consumption of food products of animal origin. Free-ranging populations of bison are managed in different ways, and are thus exposed to different health risks [8, 9]. They also occupy individual enclosures with different habitat conditions, resulting in the level of disease intensity varying between locations [11]. The occurrence of disease may be governed by environmental factors or by herd management protocols. The most convenient and common method for monitoring the occurrence of pathogens in wildlife is serological sampling [15, 16]. Samples can be relatively easy collected both *ante mortem* during routine immobilization (e.g. when putting on telemetry collars) and *post mortem* following natural death or culling.

The aim of the present study was to determine the prevalence of selected diseases known to potentially influence reproduction in the Polish bison population, *viz.* chlamydiosis, leptospirosis, Q fever, neosporosis and toxoplasmosis, with the use of a complex serological survey. The study also compares the seroprevalence of antibodies to *Chlamydia* spp., *N. caninum* and *T. gondii* among individuals kept in captivity with those from key free-ranging herds in Poland.

## Results

*Chlamydia* spp. antibodies were detected in 48 of 130 samples (36.9%). *C. burnetii* was detected in one out of 178 (0.58%), *N. caninum* in 35 out of 172 (20.3%), and *T. gondii* in 23 out of 172 (13.4%). None of the 170 samples tested for the *L. interogans* serovar Hardjo antibodies was positive (Table 1).

**Table 1:** Seroprevalence of analyzed pathogens in European bison in free ranging and captive conditions; free-ranging: *BIA* Białowieska Forest, *BOR* Borecka Forest, *KNY* Knyszyńska forest, *BIE* Bieszczady; captive, *BIA*_*c*_ Białowieża, *Ni*_*c*_ Niepołomice, *PS*_*c*_ Pszczyna, *OT*_*c*_ Other captive herds

	Free ranging				Captive			
	BIA	BOR	KNY	BIE	BIA_C_	NI_C_	PS_C_	OT_C_
*Chlamydia* spp.	10.0%	51.7%	53.3%	20.0%	6.3%	21.4%	26.3%	8.8%
*Coxiella burnetii*	10.0%	0.0%	0.0%	0.0%	0.0%	0.0%	0.0%	0.0%
*Neospora caninum*	30.0%	17.2%	33.3%	46.7%	9.4%	21.4%	0.0%	11.8%
*Toxoplasma gondii*	10.0%	20.7%	6.7%	13.3%	12.5%	0.0%	10.5%	17.6%
*Leptospira* Hardjo	0.0%	0.0%	0.0%	0.0%	0.0%	0.0%	0.0%	0.0%

Neither sex or age had any significant influence on the occurrence of antibodies to *Chlamydia* spp., *N. caninum* or *T. gondii* in European bison. All candidate models presented higher AIC values than the null model with *T. gondii*. However, location significantly differentiated the occurrence of antibodies in European bison in both of the other models, i.e. those based on *Chlamydia* spp. (p = 0.001) and *N. caninum* (p = 0.022) (Table 2).

**Table 2:** Effect of location on the occurrence of antibodies to *Chlamydia* spp. and *N. caninum* in European bison

Pathogen	Source	χ^2^	Df	*P*
*Chlamydia* spp.	Intercept	9.43	1	0.002*
	Location	23.81	7	0.002*
*N. caninum*	Intercept	0.00	1	0.999
	Location	16.33	7	0.022*

Legend: Effect of location on the occurrence of antibodies to *Chlamydia* spp. (model: χ^2^ = 31.85, df = 7, *p* < 0.001, *N* = 130) and *N. caninum* (model: χ^2^ = 33.15, df = 7, *p* < 0.001, *N* = 172) in European bison. Sex did not have a significant influence on the occurrence of *Chlamydia* spp., nor did age on *N. caninum*; therefore, these were excluded from the models in the backward elimination procedure (*statistically significant predictors)

**Fig. 1:**
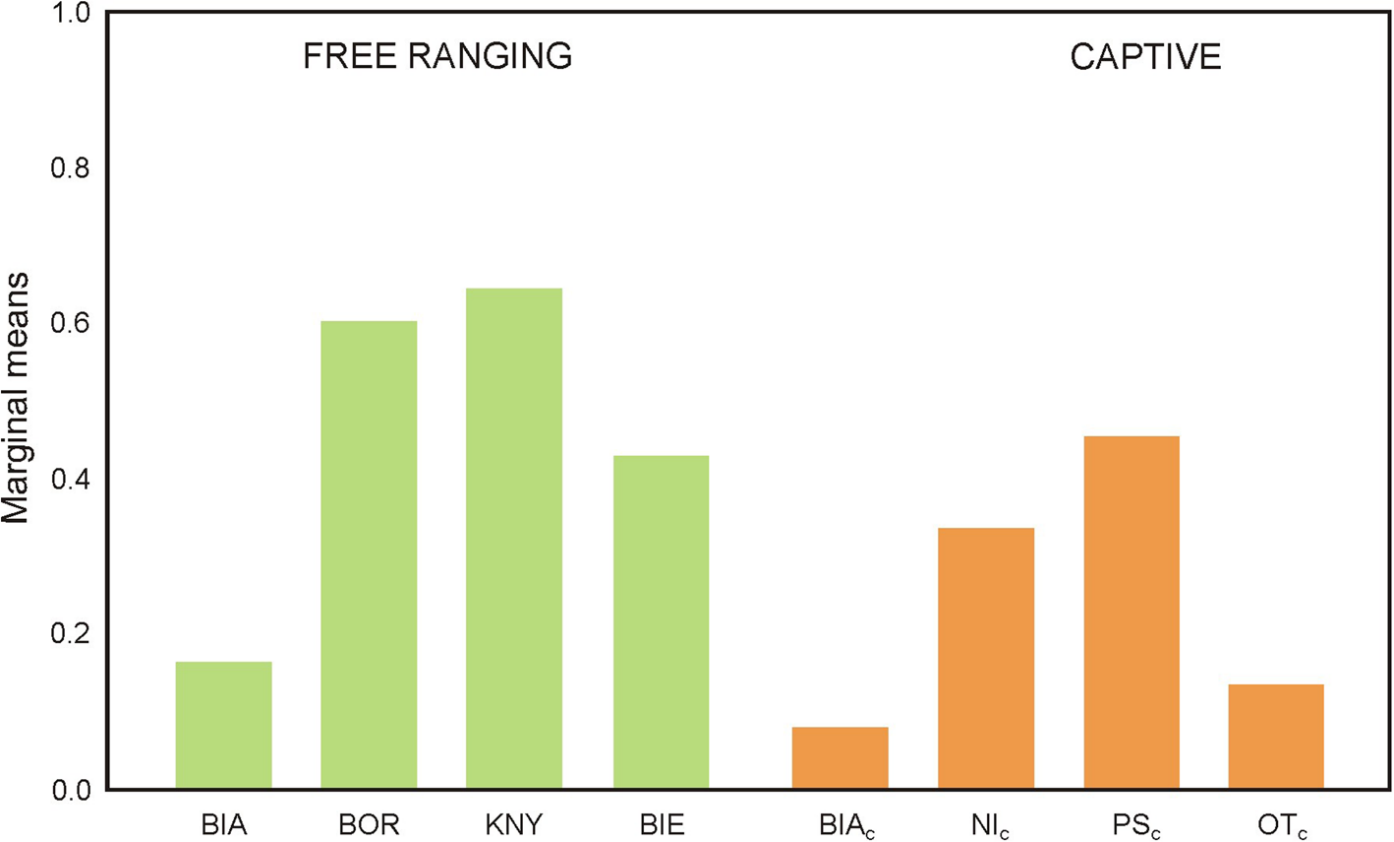
Frequency of infection by *Chlamydia* spp. in European bison from selected locations. Legend: Significant differences presented in Table S1. Free-ranging herds: BIA-Białowieska forest, BOR-Borecka forest, KNY-Kyszyńska forest, BIE-Bieszczady. Captive herds: BIA_c_ - Białowieża, Ni_c_-Niepołomice, PS_c_ - Pszczyna, OT_c_- Other captive herds

The frequency of *Chlamydia* spp. infection varied considerably with regard to location, for both the free-ranging (0.17 to 0.64) and captive herds (0.08 to 0.45) (Fig. 1, Table S1). The lowest levels were observed in the free-ranging European bison from the Białowieska Forest (BIA) and the captive herds in Białowieża (BIA_c_); however only captive herds in Białowieża (BIA_c_) and other captive herds (OT_c_) differed significantly from those observed in the free-ranging herds in the Borecka Forest (BOR) and Knyszyńska Forest (KNY).

Among the captive herds, the highest probability of infection with *Chlamydia* spp. was observed for Pszczyna (PS_c_).

**Fig. 2:**
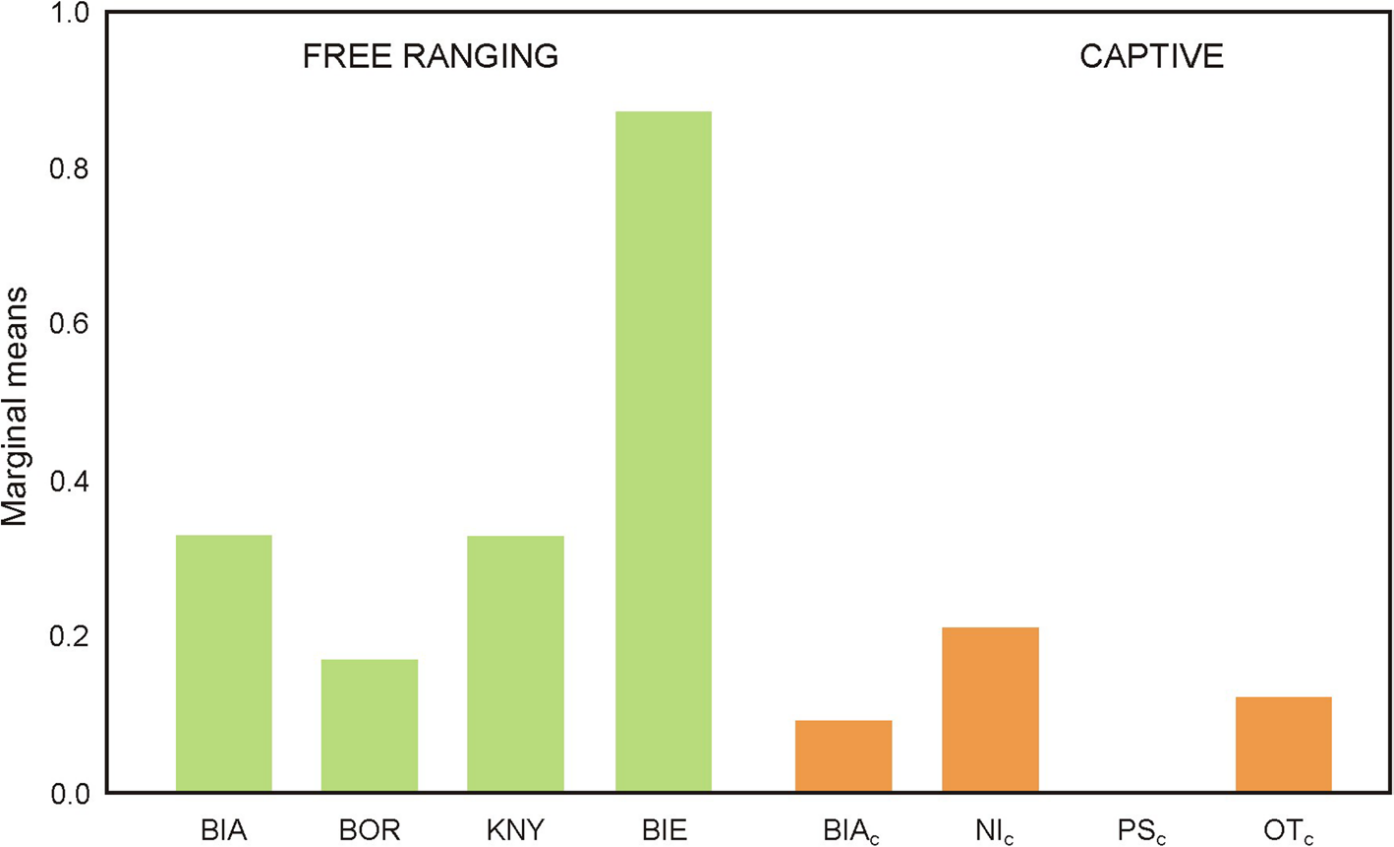
Frequency of infection with *N. caninum* in selected locations. Legend: Significant differences presented in Table S2. Free-ranging groups: BIA-Białowieska Forest, BOR-Borecka Forest, KNY-Knyszyńska forest, BIE-Bieszczady. Captive groups: BIA_c_ - Białowieża, Ni_c_- Niepołomice, PS_c_- Pszczyna, OT_c_- Other captive herds

The sero-prevalence of *N. caninum* infection differed significantly between individual locations; however, this frequency differed significantly between free-ranging herds (0.17 to 0.88) and captive ones (0.00 to 0.21) (Fig. 2, Table S2). The free-ranging European bison in the Bieszczady (BIE) herd were significantly more likely to be infected with *N. caninum* than the others, i.e. there was a significantly higher frequency of the pathogen in the population. The free-ranging herds in Knyszynska Forest (KNY) were significantly more likely to be infected than one out of the four breeding centers, *viz.* those of the Pszczyna (PS_c_).

## Discussion

Significant numbers of European bison were found to be positive for chlamydiosis (36.9%), neosporosis (20.9%) or toxoplasmosis (13.4%). Based on the known similarities between cattle and bison, these findings suggest that the European bison in these areas may be at risk of lower reproductive potential: pathogens tested in the present study are widespread, and commonly affect the reproductive system in domestic cattle [18, 26, 28]. Such data is essential for managing and protecting the European bison population, and serological monitoring can be an effective tool for further, deeper studies on this species. It should be emphasized that our findings present a fairly complete picture of the situation in the Polish European bison population, as they include individuals from four out of six free-ranging subpopulations and from 13 out of 24 captive herds.

Our findings indicate that neither sex or age had any significant effect on the occurrence of antibodies to *Chlamydia* spp., *N. caninum* or *T. gondii* in the studied European bison. They also confirm previous findings or *N. caninum* antibodies in cattle [36] or sheep [37]. However, other studies have found that both of these factors or only age was found to have a significant effect on *N. caninum* antibody levels in sheep or goats [38, 39].

Elsewhere, depending on the species, studies have found that neither sex or age had any significant effect on the occurrence of *T. gondii* antibodies [39], or that both factors were significant [40], or that only age played a role [37]. In addition, age and sex appear to be significant factors for *C. abortus* seropositivity in livestock [41]. Clearly, further research is needed to determine the effect of sex and age in individual studies.

In the present study, a high prevalence of antibodies against *Chlamydia* spp. was observed among the studied European bison (36.9%). This is in line with results obtained in other wildlife species in Spain (41.7 ± 4%) [42]. This high seroprevalence of antibodies to *Chlamydia* spp. in European bison should not be highly alarming.

While a high percentage (around 45%) of seropositive individuals has been confirmed in European bison in previous studies [16, 43], it remains unknown whether *Chlamydia* infection can influence their reproductive potential, despite the fact that it is believed to cause subclinical infections and is ubiquitous in ruminants [14]: a significantly higher seroprevalence has been noted in Polish cattle with reproductive disorders [22].

Our findings indicate that the highest probability of *Chlamydia* spp. antibodies was identified among free-ranging European bison from the Knyszyńska Forest and Borecka Forest. Among the captive herds, the highest values were found in the Pszczyna herd. Interestingly, European bison from the Knyszyńska forest are more often noticed on arable lands than those from other free-ranging herds [8]. We speculate, therefore, that the European bison visiting arable lands may also have a greater chance to come into contact with livestock, allowing the mutual transmission of bacteria [44], and numerous parasites [31] between livestock and free-living bison.

In contrast, the European bison from the Borecka Forest roam mainly in forest habitats. We speculate that health of these bison may be related to the wellbeing of those in the Pszczyna breeding center, because individuals are often transported between the two locations: the European bison in Pszczyna presented a higher probability of antibodies to *Chlamydia* spp. than other captive herds. However, further studies of the health status of the transported European bison are needed before it can be confirmed that such relocation facilitates the transmission of *Chlamydia* spp. between the herds. It is also possible that the higher probability of *Chlamydia* spp. may be linked with the presence of other wild ruminants inside the enclosure in Pszczyna, such as fallow deer (*Dama dama*), which have also demonstrated a high seroprevalance of *Chlamydia* in other countries [45].

None of the European bison were positive for *Leptospira interrogans* serovar Hardjo (*L.* Hardjo) antibodies. In contrast, they were detected in 58.3% of European bison tested in the period 1980-1983 [16] and *L. interrogans* was identified in 21.3% of European bison tested in the Białowieska Forest in the period 1991-2001 [43]. Antibodies to *Leptospira* spp. were also identified in 20 out of 240 serum samples (8.7%) taken from European bison from different regions of Poland studied in 2011-2015 by microscopic agglutination test (MAT) [15].

A positive Q fever result was obtained for one of the 178 samples (0.58%). Our results differ significantly to those from previous studies. In the period 1985-1988, extremely high seroprevalence (75.6%) was noted among a group of 47 European bison tested in Borecka Forest [46]. However, it should be stressed that such studies were conducted over 30 years ago and only within a single location, which was found to be self-limiting for Q fever. Our present findings, indicating that Q fever is not currently a threat to the European bison population, are in line with the latest reports on this topic in European bison [47]. Similarly, low prevalence levels have been recorded in other wild ruminants: 6.8% in European mouflon (*Ovis orientalis musimon*) and 2.4% in red deer (*Cervus elaphus*) in Spain [48] and 1.2% in roe deer in Flanders [49].

Positive results for *N. caninum* antibodies were obtained in 20.9% of the studied European bison. This value is higher than in previous reports from Poland, which indicate levels of 7.3% (23/320) [50] and 13% (3/23) [51]. The main transmission route of *N. caninum* is vertical in cattle, and probably also in European bison [52]; however, other transmission routes may also exist. Dogs (*Canis lupus familiaris*), coyotes (*Canis latrans*), dingoes (*Canis lupus dingo*) and gray wolves (*Canis lupus*) have been confirmed as definitive hosts [53–55]. In our studies, *N. caninum* infection was clearly more prevalent in European bison from the Bieszczady Mountains than in other subpopulations; this may be a result of the high density of the grey wolf population in this location: 9.2 individuals per 100 m^2^, compared to the mean value of 2.69 individuals per 100 m^2^ for Poland as a whole (http://www.gios.gov.pl/images/pois/monitoring-wilka-i-rysia). Wolves have been reported to hunt European bison in this location [56] and feed on European bison carcasses [57]; however, no cases have been reported of *N. caninum* in studied wolves in Poland [58].

Another reason for the atypical results observed for the Bieszczady herd is the structure of land use in the area. Following the displacement of inhabitants after WW2, this area has mainly been used for pasture [59] and, especially in the Carpathian location, shepherd dogs are commonly used to protect farm animals against wolves [60]. Hence, there is a greater possibility that European bison may come into contact with domestic dogs in this area, which can increase the degree of infection with *N. caninum*. In addition, wolves are known to often kill farm dogs in this location (Klich, unpublished data).

In the present study, 13.4% of tested European bison were positive for *T. gondii* antibodies, which is similar to the seroprevalence among cattle in Poland (13%) [61]. These results indicate that toxoplasmosis is a potential hazard for the European bison population. Antibodies to *T. gondii* were found in 24/95 (25%) of free-ranging European bison in the Białowieska Forest in the period 2008-2011 [62] and 25/240 (10.4%) of European bison from eight main breeding centers in 2011-2015 [15]. This seroprevalence may be due to environmental contamination with *T. gondii* oocysts shed by domestic and wild felids [63]. Although there is currently no evidence that *T. gondii* infection has any clinical consequences in European bison, it may nevertheless represent a potential threat to reproduction, particularly since transplacental transmission has been confirmed: *T. gondii* genetic material has been isolated from the brain of an aborted European bison fetus from Bialowieza National Park [64]. Studies have noted high levels of *T. gondii* seropositivity in Polish wildlife: 21.4% of red deer, 37.6% of wild boar (*Sus scrofa*), and 30.4% of roe deer [65].

The present study has some limitations. Firstly, the ELISA tests used for detection are not specifically intended for use in European bison samples. However, such tests are commonly used for serological monitoring in domestic cattle, which are closely related to European bison. Even so, to obtain greater sensitivity and specificity in future studies on European bison, further test optimization and cut-off adaptation is required based on larger numbers of samples. In addition, the tests examined the presence of IgG antibodies, which can only indicate contact with the pathogen; therefore, the results only indicate the distribution of the studied pathogens in European bison. Although this is still an important finding, future studies should confirm any serological findings with direct detection methods such as PCR. Nevertheless, despite these limitations, our findings can play an important role in recognizing current health threats and identifying the pathogens that commonly infect European bison in Poland; this is particularly important considering the small size of the European bison population and its endangered status.

## Conclusion

Our findings indicate that existing Polish European bison herds have had contact with the etiological agents of chlamydiosis, neosporosis and toxoplasmosis while no Q fever was observed. Contrary to previous reports, no antibodies to *L.* Hardjo were detected. The seroprevalence of *Chlamydia* spp. in European bison depends on location, with the free-ranging herds in Knyszyńska Forest and Borecka Forest, and captive ones in Pszczyna being the most exposed; therefore, serological monitoring should be continued, especially in those populations. The free-ranging herds also demonstrated a greater prevalence of *N. caninum* antibodies than the enclosed-breeding herds, and the highest exposure was observed in the subpopulation from the Bieszczady Mountains; this is probably associated with the high number of definitive hosts, *viz.* grey wolves and dogs, in this area.

We suggest that further serological monitoring should be performed, together with more specific microbiological studies, especially in case of abortions, to confirm the significance of the studied pathogens on European bison propagation.

## Methods

### Material collection

**Fig. 3:**
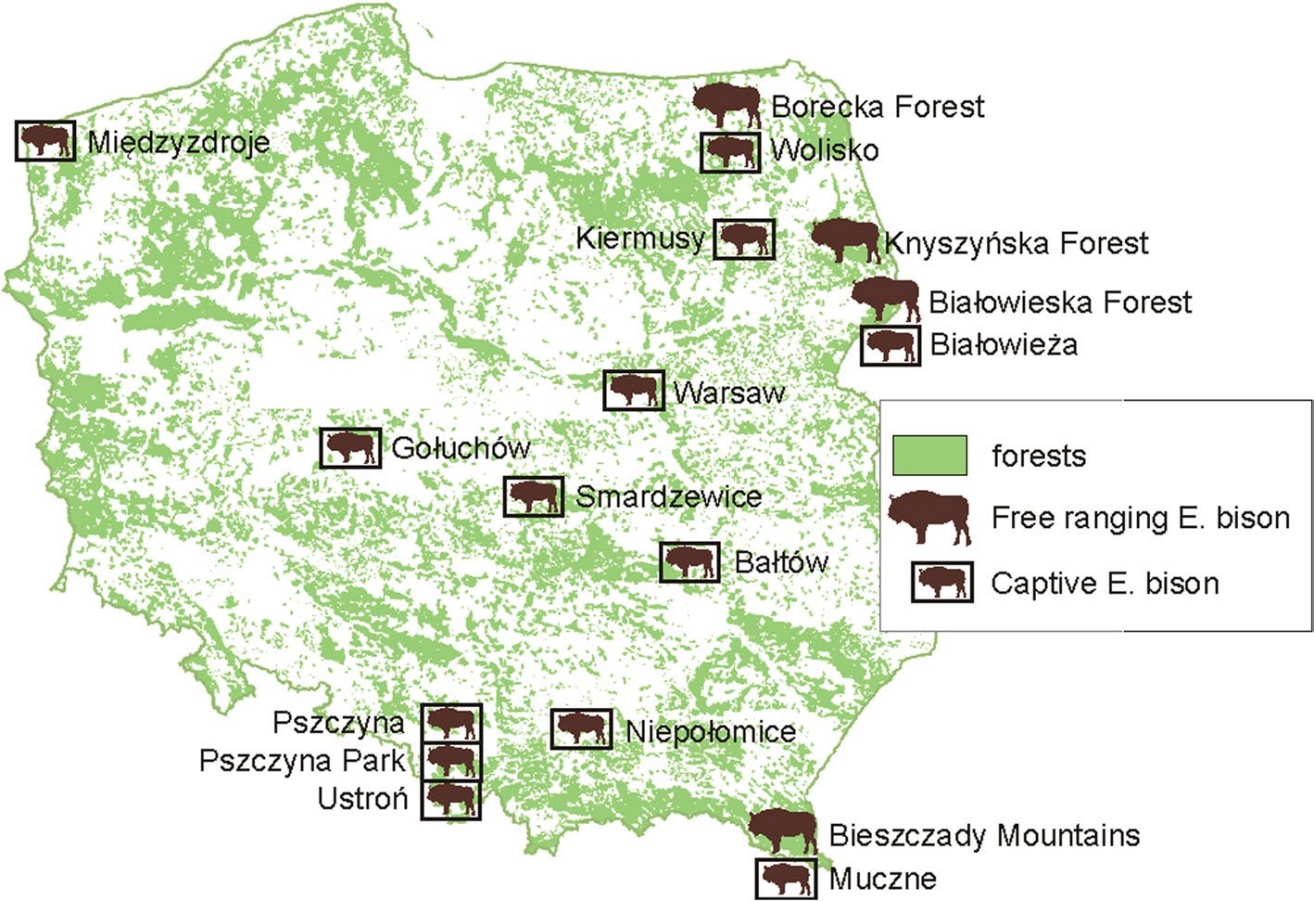
Location of blood sample collection from captive and free ranging herds of European bison in Poland. The figure belongs to the authors of the publication

Blood samples from 183 European bison were collected between September 2017 and December 2019 from 17 locations; four free-ranging and 13 captive herds (Fig. 3). Among the free-ranging herds, blood samples were collected from the following locations: Białowieska Forest (*n* = 10), Borecka Forest (*n* = 29), Knyszyńska Forest (*n* = 30) and Bieszczady Mountains (*n* = 15). Among the captive herds, the samples were collected from Białowieża (*n* = 32), Niepołomice (*n* = 14), Pszczyna (*n* = 19), and various other captive herds (Bałtów, Gołuchów, Kiermusy, Międzyzdroje, Muczne, Pszczyna Park, Smardzewice, Ustroń, Warsaw, Wolisko) (*n* = 34). The age of the tested European bison ranged from 0.25 (three months) to 25 years with a median value of four years. Samples were collected *ante mortem* (*n* = 106) and *post mortem* (*n* = 75). Due to the fact the bison is a strictly protected species, the collection of material could only take place during other activities related to the management of the population (e.g. immobilization before transport or placing a telemetric collar, death of animal). Samples were collected whenever there was such possibility (by qualified veterinarians during immobilisation or from dead animals).

As *ante-mortem* sampling was conducted during immobilisation as part of standard veterinary care, the procedure did not require approval according to II Local Ethical Committee For Animal Experiments in WULS-SGGW in Warsaw. Immobilisation was conducted as described previously [66].

Collection and storage of serum samples from dead animals was based on the decision of Regional Director of Environmental Protection in Warsaw. According to the decision, collection of dead animals for scientific purposes does not need any permit as described previously [8]. Some animals were found dead or were culled for health reasons such as severe infectious disease or limb fracture, among others. Among the 23 female European bison for which autopsy protocols were available, only five presented lesions concerning the reproductive system (e.g. serous or purulent discharge from the vagina, vaginal congestion, pyometritis); this small number did not allow for any statistical comparison of the presence of lesions and antibodies.

The blood was obtained from the jugular or tail vein during both *ante-mortem and post-mortem* collection, and sometimes from the heart, in the case of dead animals. The samples were collected into 10 ml sterile tubes with clot activator; these were transported to the laboratory at 4 °C and then centrifuged to obtain serum. Serum samples were frozen and stored at -20 °C for further analysis.

### Serological tests

After defrosting at room temperature, the serum samples were tested with single-well indirect enzyme-linked immunosorbent assay (ELISA) kits: PrioCHECK™ Ruminant *Chlamydophila spp.* Ab Kit (Thermofisher, US) (*n* = 130), PrioCHECK™ Ruminant Q Fever Ab Plate Kit (Thermofisher, US) (*n* = 178), PrioCHECK™ *L.* Hardjo Ab Plate Kit (Thermofisher, US) (*n* = 170), PrioCHECK™ Bovine *Neospora* Ab 2.0 Serum/Milk Kit (Thermofisher, US) (*n* = 172), Vetline *Toxoplasma* ELISA KIT (Ingenasa, Spain) (*n* = 172). Different numbers of samples were used for each test as insufficient serum was collected from some of the tested European bison.

The conducted tests detect specific antibodies to *Chlamydia* spp*.*, *Coxiella burnetii, Leptospira interogans* serovar Hardjo, *Neospora caninum* and *Toxoplasma gondii*. The first two tests are dedicated to cattle, sheep and goats, the next two are solely for cattle, and the last one is a multispecies test. All tests were conducted in accordance with the manufacturer’s instructions. Results were read at a wavelength of 450 nm with an EPOCH spectrophotometer (BioTek Instruments Inc., US) and calculated in accordance with the manufacturer’s instructions as for cattle.

Briefly, in the PrioCHECK™ Ruminant *Chlamydophila spp.* Ab Kit (Thermofisher, US) samples and controls were distributed to antigen-coted plate and any specific antibodies were bound to the antigen. Then plate was washed, and an anti-ruminant conjugate was added. The unbound conjugate was then washed out and chromogenic substrate was added. After stopping the reaction, the intensity of the resulting yellow colour, measured with a spectrophotometer, was proportional to the level of specific antibodies present in each well.

In the PrioCHECK™ Ruminant Q Fever Ab Plate Kit (Thermofisher, US) and PrioCHECK™ *L.* Hardjo Ab Plate Kit (Thermofisher, US), the serum was dispensed to the inactivated-antigen coated plate and antibodies were bound during incubation. The antibodies were then detected using an anti-bovine monoclonal antibody conjugated to horseradish peroxidise enzyme. The bound conjugate was visualised by incubation with Chromogen (TMB) Substrate. After stopping the reaction, the intensity of colour was measured with a spectrophotometer.

For the PrioCHECK™ Bovine *Neospora* Ab 2.0 Serum/Milk Kit (Thermofisher, US), the samples were distributed and washed, and an anti-bovine conjugate labelled with peroxidase (HRP) was added and bound to the antibodies joined with the antigens on the plate. The plate was washed, and a chromogenic substrate and stopping solution were added. After stopping, the intensity of the resulting yellow colour, measured with a spectrophotometer, was proportional to level of the specific antibodies in the sample. A similar principle was used in the Vetline *Toxoplasma* ELISA KIT (Ingenasa, Spain).

### Statistical analysis

The occurrence of antibodies to *Chlamydia* spp., *N. caninum* and *T. gondii* was analyzed statistically. A separate model was built for each pathogen, where the presence of antibodies for a given pathogen was analyzed as the dependent binary variable: the number 1 indicated present, and 0 absent. A similar set of independent variables was applied: age (years) and sex of animal, and location of the sample collection. The location of sample collection was assigned eight categories based on the sampled herd: the free-ranging herds from the Białowieska Forest, Borecka Forest, Knyszyńska Forest and Bieszczady were assigned four categories, the captive herds from Białowieża, Pszczyna and Niepołomice were assigned three, and the other captive herds with limited sample numbers were combined into one.

The data was analyzed using a generalized linear model with binomial distribution and logit link function [67]. The best fitting model was selected using a backward elimination procedure with an AIC value comparison. The statistical analyses were performed using SPSS.

As not all the blood samples were tested for each pathogen, the numbers of observations differed between models.

## Supplementary Information

**Additional file 1: Table S1.** Significant differences in frequency of infection with *Chlamydia* spp. in European bison in free-ranging and captive conditions: Free-ranging: BIA – Białowieska Forest, BOR – Borecka Forest, KNY – Knyszyńska forest, BIE – Bieszczady; Captive: BIA_c_ – Białowieża, Ni_c_ – Niepołomice, PS_c_ – Pszczyna, OT_c_ – Other captive herds. Significant differences (*p* < 0.05) are presented for pairwise comparisons with Sidak correction.

**Additional file 2: Table S2.** Significant differences in frequency of infection with *N. caninum* in European bison in free-ranging and captive conditions: Free-ranging: BIA – Białowieska Forest, BOR – Borecka Forest, KNY – Knyszyńska forest, BIE – Bieszczady; Captive: BIA_c_ – Białowieża, Ni_c_ – Niepołomice, PS_c_ – Pszczyna, OT_c_ – Other captive herds. Significant differences are presented (*p* < 0.05) as pairwise comparisons with Least Significant Difference.

## Data Availability

The datasets used and/or analysed during the current study are available from the corresponding author on reasonable request.

## Abbreviations

BTB: Bovine tuberculosis
BIA: Białowieska Forest
BIE: Bieszczady Mountains
BIA_c_: Białowieża (captive)
BOR: Borecka Forest
KNY: Knyszyńska Forest
OT-C: Other enclosures (captive)
NI_c_: Niepołomice (captive)
PS_c_: Pszczyna (captive)
