# Supplementary material for: Pathogens with potential impact on reproduction in captive and free-ranging European bison (Bison bonasus) in Poland - a serological survey
Source: BMC Vet Res. 2021 Nov 4;17:345. doi: 10.1186/s12917-021-03057-8 (PMC8567710; doi:10.1186/s12917-021-03057-8)
Supplement: Supplementary file 2 — Additional file 2: Table S2. Significant differences in frequency of infection with N. caninum in European bison in free-ranging and captive conditions: Free-ranging: BIA – Białowieska Forest, BOR – Borecka Forest, KNY – Knyszyńska forest, BIE – Bieszczady; Captive: BIAc – Białowieża, Nic – Niepołomice, PSc – Pszczyna, OTc – Other captive herds. Significant differences are presented (p < 0.05) as pairwise comparisons with Least Significant Difference. [file 12917_2021_3057_MOESM2_ESM.docx]

Table. S2. Significant differences in frequency of infection with *N. caninum* in European bison in free-ranging and captive conditions: Free-ranging: BIA – Białowieska Forest, BOR – Borecka Forest, KNY – Knyszyńska forest, BIE – Bieszczady; Captive: BIA_c_ – Białowieża, Ni_c_ – Niepołomice, PS_c_ – Pszczyna, OT_c_ – Other captive herds. Significant differences are presented (p<0.05) as pairwise comparisons with Least Significant Difference.

|  | Free ranging | | | | Captive | | | |
| --- | --- | --- | --- | --- | --- | --- | --- | --- |
|  | BIA | BOR | KNY | BIE | BIA_C_ | NI_C_ | PS_C_ | OT_C_ |
| BIA |  |  |  |  |  |  |  |  |
| BOR |  |  |  | P<0.05 |  |  |  |  |
| KNY |  |  |  | P<0.05 |  |  | P<0.05 |  |
| BIE |  | P<0.05 | P<0.05 |  | P<0.05 | P<0.05 | P<0.05 | P<0.05 |
| BIA_C_ |  |  |  | P<0.05 |  |  |  |  |
| NI_C_ |  |  |  | P<0.05 |  |  |  |  |
| PS_C_ |  |  | P<0.05 | P<0.05 |  |  |  |  |
| OT_C_ |  |  |  | P<0.05 |  |  |  |  |
